# Supplementary material for: Tropical extreme droughts drive long-term increase in atmospheric CO2 growth rate variability
Source: Nat Commun. 2022 Mar 7;13:1193. doi: 10.1038/s41467-022-28824-5 (PMC8901933; doi:10.1038/s41467-022-28824-5)
Supplement: Supplementary file 1 — Supplementary Information [file 41467_2022_28824_MOESM1_ESM.pdf]

Supplementary Materials

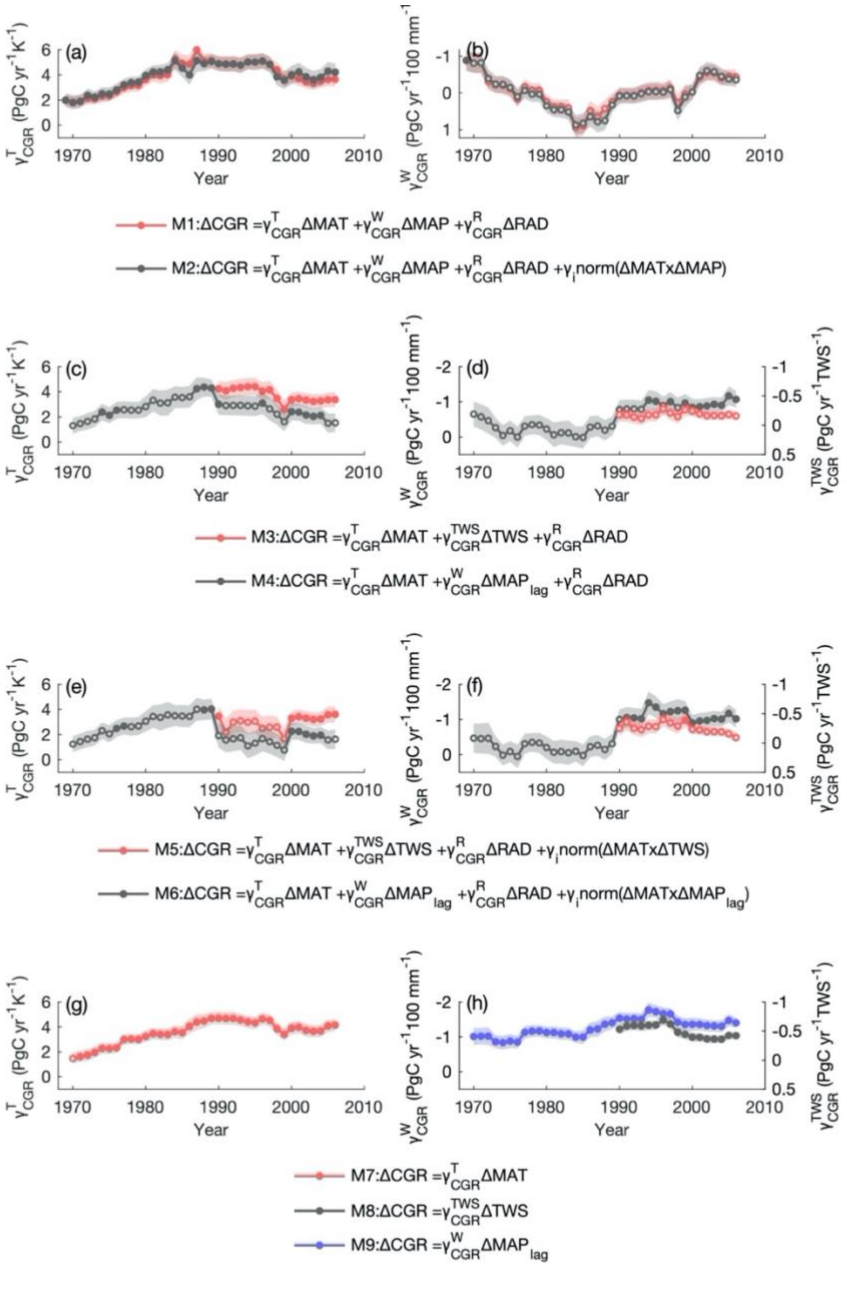

Supplementary Figure 1 | Temperature sensitivity ($\gamma_{\mathrm{CGR}}^{\mathrm{T}}$) and water sensitivity ($\gamma_{\mathrm{CGR}}^{\mathrm{W}}$ or $\gamma_{\mathrm{CGR}}^{\mathrm{TWS}}$) of CGR derived from nine competing methods (M1-M9). Left panels show the temperature sensitivity and right panels show the water sensitivity, with the details of models shown in the legend. All climate sensitivities are calculated using 20 moving windows from 1959 to 2016. Shaded areas of indicate one standard deviation of climate sensitivities derived from 100 bootstrap estimates, considering the CGR

uncertainty of 0.2 PgC yr^{-1} [1]. A solid circle marker indicates significant correlation ($p < 0.05$) between the climate variable and ΔCGR in that 20-year window, while open circle indicates non-significant correlation.

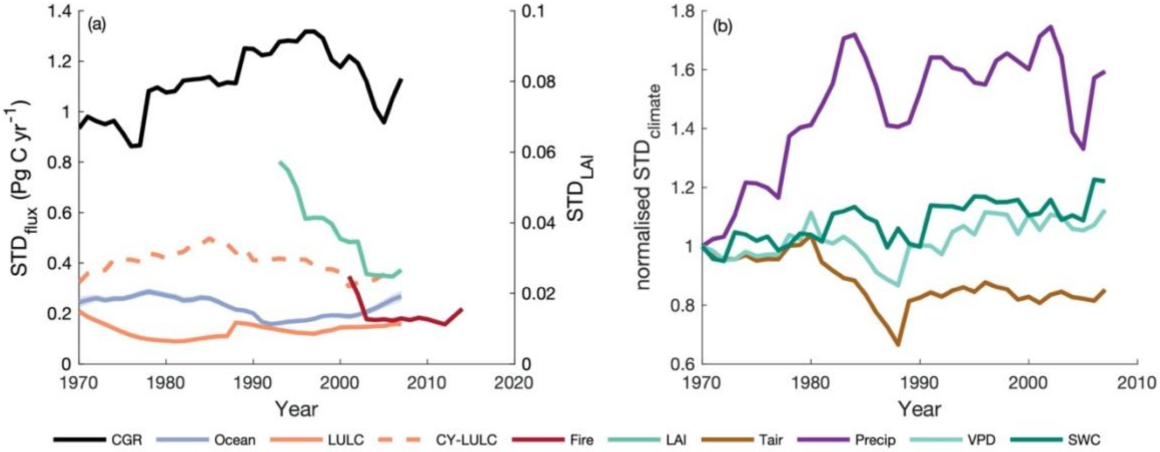

Supplementary Figure 2 | Temporal changes in the magnitude of the interannual variability of CGR, ocean uptake, land use change emissions, fire emissions, leaf area index (LAI) and climate variables. Interannual variability is indicated by one standard deviation of annual values (STD) within 20-year moving window. (a) CGR, ocean carbon uptake and land use change emissions are provided by the Global Carbon Budget. CY-LULC indicates the land use change emissions estimated by ORCHIDEE, which have considered the changes in carbon density and climate variability for the land use change emissions[2]. Fire emissions are obtained from Global Fire Emissions Database (GFED4s). Since fire emissions record only range from 1997 to 2020, we used 10-year moving window to calculate the STD of fire fluxes. The shadings of ocean uptake indicate one standard error of eight models. LAI is the GMMIS AVHRR3g LAI over the tropics from 1982 to 2016. (b) STD of air temperature (Tair), precipitation (Precip), vapor pressure deficit (VPD) and soil water content (SWC) are normalised by the STD of first 20-year window (1959-1979) to make them comparable.

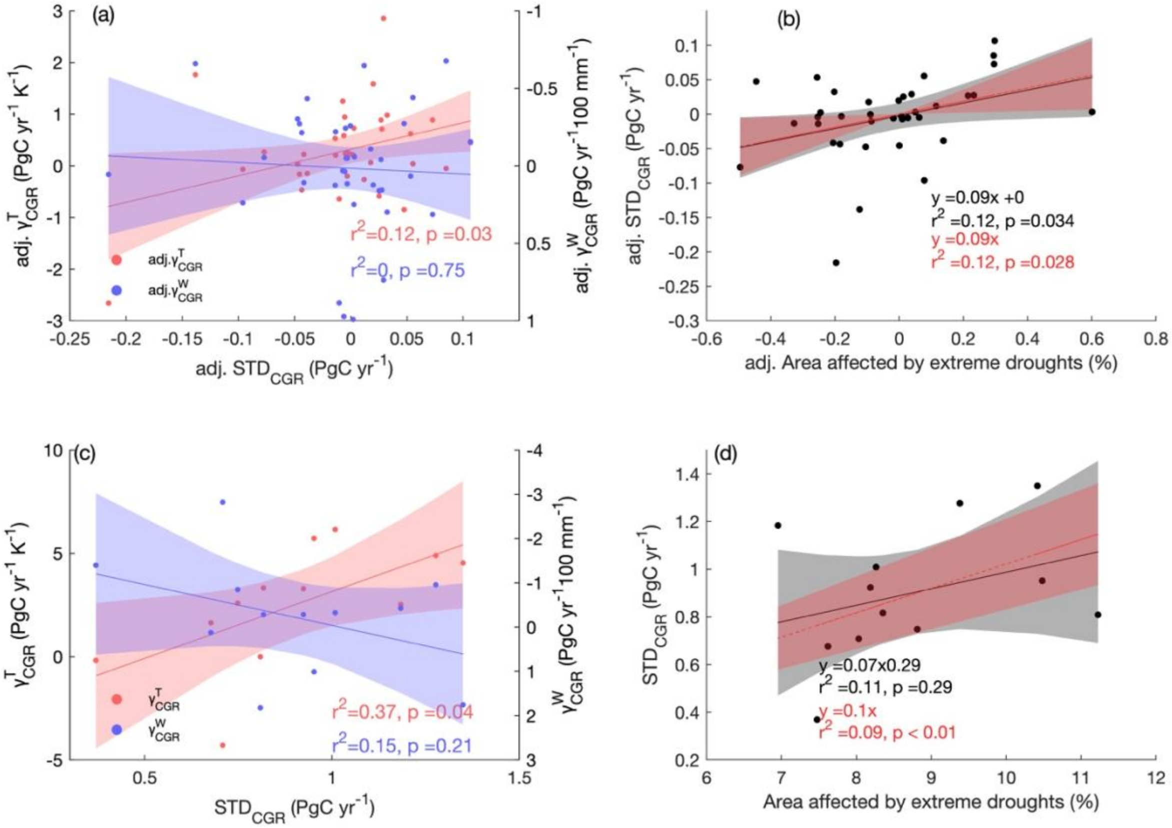

Supplementary Figure 3 | Remove the autocorrelations in Fig. 1d and Fig. 2c. We used two methods to evaluate the impact of autocorrelation. In (a) and (b), we used the Cochrane-Ocrutt procedure (see Methods) to remove autocorrelations in time series and obtain adjusted climate sensitivities of CGR (i.e., adj. $\gamma^{\mathrm{T}}_{\mathrm{CGR}}$ and adj. $\gamma^{\mathrm{W}}_{\mathrm{CGR}}$) and adjusted STD_{CGR}. In (c) and (d), we divided the time series of CGR into 12 independent 5-year segments and derived $\gamma^{\mathrm{T}}_{\mathrm{CGR}}$, $\gamma^{\mathrm{W}}_{\mathrm{CGR}}$ and STD_{CGR} for each 5-year segment to obtain non-autocorrelated time series of climate sensitivities and STD_{CGR}. (a,c) The relationships between climate sensitivities of CGR and STD_{CGR} (shading: 95% confidence interval); (b) The relationship between STD_{CGR} and extreme drought-affected area (shading: 95% confidence interval).

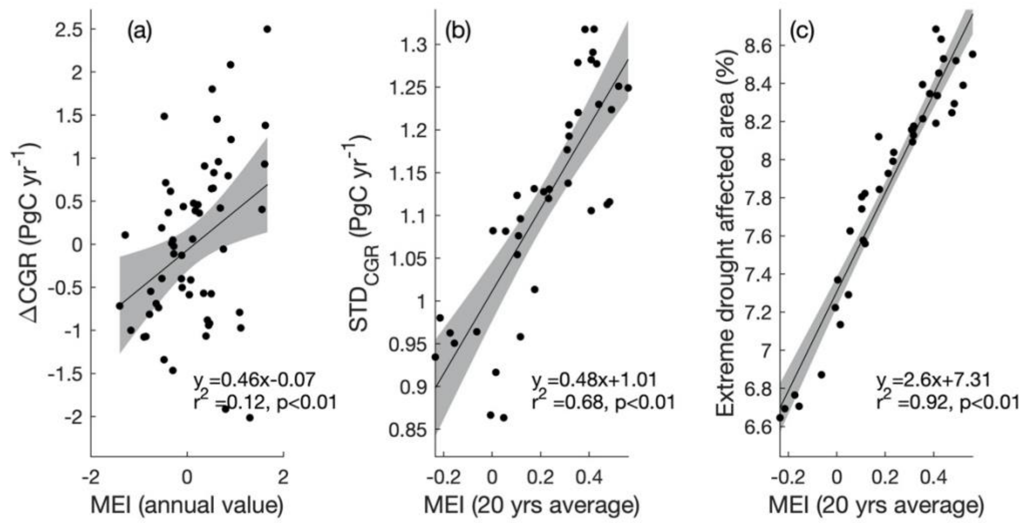

Supplementary Figure 4 | ENSO effects on annual CGR anomalies (ΔCGR), STD_{CGR} and tropical extreme drought-affected area. Multivariate ENSO Index (MEI; https://psl.noaa.gov/enso/mei.old/table.html) is used to indicate ENSO phases, where positive values mean warm, El Niño events. Shadings indicate 95% confidence intervals.

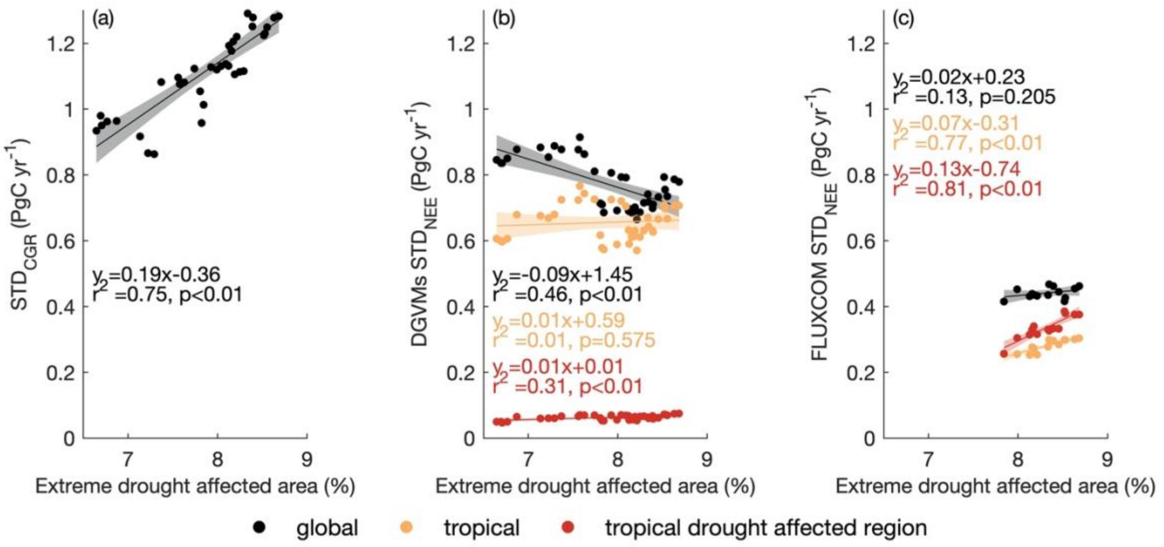

Supplementary Figure 5 | The correlations between STD_{CGR} (STD_{NEE}) and extreme drought-affected area. STD_{NEE} were calculated based on either global annual NEE, tropical annual NEE or tropical drought affected regions' annual NEE. Annual NEE were obtained from DGVMs and FLUXCOM. We used the ensemble mean NEE from each group of models. Shadings indicate 95% confidence intervals.

Supplementary Table 1. The dynamic global vegetation models (DGVMs) from TRENDY v6.

Model	Reference
CABLE	Haverd et al. (2018)[3]
CLASS-CTEM	Melton and Arora (2016)[4]
CLM4.5(BGC)	Oleson et al. (2013)[5]
DLEM	Tian et al. (2015)[6]
ISAM	Jain et al. (2013)[7]
JSBACH	Reick et al. (2013)[8]
JULES	Clark et al. (2011)[9]
LPJ-GUESS	Smith et al. (2014)[10]
LPJ	Sitch et al. (2003)[11]
LPX-Bern	Keller et al. (2017)[12]
OCN	Zaehle and Friend (2010)[13]
ORCHIDEE	Krinner et al. (2005)[14]
ORCHIDEE-MICT	Guimberteau et al. (2018)[15]
SDGVM	Woodward et al. (1995)[16]
VISIT	Kato et al. (2013)[17]

Supplementary References

1. Le Quéré, C. *et al.* Global Carbon Budget 2018. *Earth Syst. Sci. Data* **10**, 2141–2194 (2018).
2. Yue, C., Ciais, P., Houghton, R. A. & Nassikas, A. A. Contribution of land use to the interannual variability of the land carbon cycle. *Nat. Commun.* **11**, 3170 (2020).
3. Haverd, V. *et al.* A new version of the CABLE land surface model (Subversion revision r4601) incorporating land use and land cover change, woody vegetation demography, and a novel optimisation-based approach to plant coordination of photosynthesis. *Geosci. Model Dev.* **11**, 2995–3026 (2018).
4. Melton, J. R. & Arora, V. K. Competition between plant functional types in the Canadian Terrestrial Ecosystem Model (CTEM) v. 2.0. *Geosci. Model Dev.* **9**, 323–361 (2016).
5. Oleson, K. W. *et al.* Technical Description of version 4.5 of the Community Land Model (CLM). (2013).
6. Tian, H. *et al.* North American terrestrial CO2 uptake largely offset by CH4 and N2O emissions: toward a full accounting of the greenhouse gas budget. *Clim. Change* **129**, 413–426 (2015).
7. Jain, A. K., Meiyappan, P., Song, Y. & House, J. I. CO2emissions from land-use change affected more by nitrogen cycle, than by the choice of land-cover data. *Glob. Chang. Biol.* **19**, 2893–2906 (2013).

8. Reick, C. H., Raddatz, T., Brovkin, V. & Gayler, V. Representation of natural and anthropogenic land cover change in MPI-ESM. *J. Adv. Model. Earth Syst.* **5**, 459–482 (2013).
9. Clark, D. B. *et al.* The Joint UK Land Environment Simulator (JULES), model description – Part 2: Carbon fluxes and vegetation dynamics. *Geosci. Model Dev.* **4**, 701–722 (2011).
10. Smith, B. *et al.* Implications of incorporating N cycling and N limitations on primary production in an individual-based dynamic vegetation model. *Biogeosciences* **11**, 2027–2054 (2014).
11. Sitch, S. *et al.* Evaluation of ecosystem dynamics, plant geography and terrestrial carbon cycling in the LPJ dynamic global vegetation model. *Glob. Chang. Biol.* **9**, 161–185 (2003).
12. Keller, K. M. *et al.* 20th century changes in carbon isotopes and water-use efficiency: Tree-ring-based evaluation of the CLM4.5 and LPX-Bern models. *Biogeosciences* **14**, 2641–2673 (2017).
13. Zaehle, S. & Friend, A. D. Carbon and nitrogen cycle dynamics in the O-CN land surface model: 1. Model description, site-scale evaluation, and sensitivity to parameter estimates. *Global Biogeochem. Cycles* **24**, 1–13 (2010).
14. Krinner, G. *et al.* A dynamic global vegetation model for studies of the coupled atmosphere-biosphere system. *Global Biogeochem. Cycles* **19**, 1–33 (2005).
15. Guimberteau, M. *et al.* ORCHIDEE-MICT (v8.4.1), a land surface model for the high latitudes: model description and validation. *Geosci. Model Dev.* **11**, 121–163 (2018).
16. Woodward, F. I., Smith, T. M. & Emanuel, W. R. A global land primary productivity and phytogeography model. *Global Biogeochem. Cycles* **9**, 471–490 (1995).
17. Kato, E., Kinoshita, T., Ito, A., Kawamiya, M. & Yamagata, Y. Evaluation of spatially explicit emission scenario of land-use change and biomass burning using a process-based biogeochemical model. *J. Land Use Sci.* **8**, 104–122 (2013).
